# Multi-drug resistant *Vibrio cholerae* O1 variant El Tor isolated in northern Vietnam between 2007 and 2010

**DOI:** 10.1099/jmm.0.034744-0

**Published:** 2012-03

**Authors:** Huu Dat Tran, Munirul Alam, Nguyen Vu Trung, Nguyen Van Kinh, Hong Ha Nguyen, Van Ca Pham, Mohammad Ansaruzzaman, Shah Manzur Rashed, Nurul A. Bhuiyan, Tuyet Trinh Dao, Hubert P. Endtz, Heiman F. L. Wertheim

**Affiliations:** 1 National Hospital of Tropical Diseases, 78 Giai Phong Street, Hanoi, Vietnam; 2 Department of Medical Microbiology, Hanoi Medical University, 1 Ton That Tung Street, Hanoi, Vietnam; 3 Enteric & Food Microbiology Laboratory, ICDDR,B: International Centre for Diarrhoeal Disease Research, Bangladesh GPO Box 128, Dhaka 1000, Bangladesh; 4 Department of Infectious Diseases, Hanoi Medical University, 1 Ton That Tung Street, Hanoi, Vietnam; 5 Department of Medical Microbiology & Infectious Diseases, Erasmus MC, University Medical Centre Rotterdam, The Netherlands; 6 Oxford University Clinical Research Unit Vietnam, Wellcome Trust Major Overseas Program, National Hospital of Tropical Diseases, 78 Giai Phong Road, Dong Da, Hanoi, Vietnam; 7 Centre for Tropical Medicine, Nuffield Department of Clinical Medicine, University of Oxford, Churchill Hospital, Old Road, Oxford OX3 7LJ, UK

## Abstract

Since 2007, there has been a re-emergence of cholera outbreaks in northern Vietnam. To understand the molecular epidemiological relatedness and determine the antibiotic susceptibility profiles of responsible *V. cholerae* O1 outbreak strains, a representative collection of 100 *V. cholerae* O1 strains was characterized. *V. cholerae* O1 strains isolated from diarrhoeal patients in northern Vietnam between 2007 and 2010 were investigated for antibiotic susceptibility and characterized by using phenotypic and genotypic tests, including PFGE analysis. Ten clinical *V. cholerae* O1 isolates from Bangladesh and Zimbabwe were included for comparison. The results revealed that all isolates were resistant to co-trimoxazole and nalidixic acid, 29 % were resistant to tetracycline and 1 % were resistant to azithromycin. All strains were susceptible to ampicillin–sulbactam, doxycycline, chloramphenicol and ciprofloxacin and 95 % were susceptible to azithromycin. MIC values did show reduced susceptibility to fluoroquinolones and 63 % of the strains were intermediately resistant to tetracycline. The isolates expressed phenotypic traits of both serogroup O1 Ogawa and El Tor and harboured an *rstR* El Tor and *ctx*B classical biotype. Among the outbreak isolates, only a single PFGE pattern was observed throughout the study period. This study shows that multi-drug resistant *V. cholerae* altered El Tor producing classical CT strains are now predominant in northern Vietnam.

## Introduction

Cholera is an acute diarrhoeal disease, causing profuse watery stools, which, if untreated, leads to rapid dehydration and death (Kaper *et al.*, 1995). Cholera is prevalent in developing countries with inadequate sanitation and poor access to safe drinking water (Zuckerman *et al.*, 2007). From 1995 to 2005, approximately 100 000–300 000 cases of cholera causing 1500 deaths were reported annually to the WHO (Griffith *et al.*, 2006). Cholera is caused by the Gram-negative bacterium *Vibrio cholerae* serogroups O1 and O139 (Kaper *et al.*, 1995). Serogroup O1 can be categorized into two biotypes (classical or El Tor), and three serotypes (Ogawa, Inaba or Hikojima) (Sack *et al.*, 2004). The seventh, and current, pandemic of cholera is being caused by the El Tor biotype while the fifth and sixth pandemics of cholera were caused by the classical biotype (Alam *et al.*, 2010). Recently, new variants of *V. cholerae* O1 have emerged and spread throughout many countries of Asia, Africa and America that display a mixture of phenotypic and genotypic traits of both classical and El Tor biotypes (Alam *et al.*, 2010; Ansaruzzaman *et al.*, 2007).

Since the first variants of *V. cholerae* O1 El Tor were identified in Bangladesh, several other atypical El Tor strains have been identified and reported (Ansaruzzaman *et al.*, 2007; Choi *et al.*, 2010). Genotypic tests to differentiate *V. cholerae* O1 biotypes include *ctxB* (cholera toxin B), *rstR* (repeat sequence transcriptional) and *tcpA* (toxin-coregulated pili) gene typing (Safa *et al.*, 2010). The El Tor strains that cause cholera in many countries have shifted from genotype 3 (found in El Tor strains from the seventh pandemic and the Latin American epidemic) to genotype 1 (found in strains of classical biotype worldwide and US Gulf Coast El Tor strains). In addition, atypical El Tor strains of *V. cholerae* O1 from Asia and Africa harboured an *rstR* allele known as *rstR*^Classical^, which corresponds with CTXΦ^Classical^ and are reported as hybrid strains of the El Tor biotype (Ansaruzzaman *et al.*, 2004). Moreover, the multi-drug resistant *V. cholerae* O1 altered El Tor has emerged as major problem in many countries in Asia and Africa (Ang *et al.*, 2010; Jain *et al.*, 2011; Kumar *et al.*, 2010; Quilici *et al.*, 2010).

In 2007, a cholera outbreak emerged in northern Vietnam, where only a few cases of cholera had previously been reported (Dalsgaard *et al.*, 1999). The strains of *V. cholerae* O1 responsible for this outbreak were variants of the El Tor biotype displaying combinations of classical and El Tor features (Nguyen *et al.*, 2009). New cholera cases continued to be detected in northern Vietnam until 2010, especially in Hanoi. The *V. cholerae* O1 strains associated with cholera outbreaks between 2007 and 2010 in northern Vietnam have not yet been characterized and their susceptibility to antimicrobials has not been reported. Moreover, there is lack of information about the genetic diversity among *V. cholerae* O1 strains isolated from patients in northern Vietnam between 2007 and 2010.

In the present study, we investigated the antimicrobial susceptibilities and the molecular epidemiological traits of *V. cholerae* O1 strains isolated in northern Vietnam from 2007 to 2010. *V. cholerae* O1 strains from Bangladesh and Zimbabwe were also included for comparison.

## Methods

### 

#### Bacterial strains.

In this study, we combined two collections of *V. cholerae* O1 strains that were selected randomly from diarrhoeal patients admitted to the National Hospital for Tropical Diseases (NHTD), a tertiary-care hospital in Hanoi, Vietnam. The NHTD served as a referral centre during the cholera outbreaks in Hanoi and the surrounding areas between 2007 and 2010. Antimicrobial susceptibility testing was performed on 100 isolates from patients admitted in 2008. A second collection contained 100 randomly selected strains isolated from patients with acute diarrhoea who were admitted to the NHTD between 2007 and 2010 (25 isolates for each year). *V. cholerae* was identified and confirmed by using standard biochemical methods (Islam *et al.*, 2009). Serological identification of isolates was done by performing slide agglutination tests with commercially available polyvalent antiserum against *V. cholerae* O1 and monovalent antiserum against *V. cholerae* O1 serogroups Ogawa and Inaba (Bio-Rad) (Dalsgaard *et al.*, 1999). The second collection was shipped in soft agar at ambient temperature to the ICDDR,B for phenotypic and genotypic testing and PFGE analysis. Reference strains for each of the *V. cholerae* O1 biotypes were used: classical (O395) and El Tor (N16961). Ten clinical isolates each of *V. cholera* O1 from Bangladesh and Zimbabwe were included for comparison.

#### Serotyping and biotyping.

Vietnamese *V. cholerae* strains were serotyped by using the slide agglutination test with specific polyvalent antiserum to *V. cholerae* O1 and serotype specific antisera to Inaba and Ogawa from the ICDDR,B (Ansaruzzaman *et al.*, 2004). For biotype analysis, we used chicken erythrocyte agglutination, sensitivity to polymycin B and Mukerjee classical phage IV and Mukerjee El Tor phage 5 tests (Kaper *et al.*, 1995; Sack *et al.*, 2004).

#### PCR assays.

The characterization of biotype and genotype were performed with molecular methods. Species-specific identification of *V. cholerae* was carried out by using *V. cholerae* species-specific *ompW* PCR (Nandi *et al.*, 2000). The serogroups of these strains were reconfirmed by multiplex PCR targeted to identify genes encoding O1 (*rfb*) and O139 (*rfb*), specific O biosynthetic genes and the cholera toxin (CTX) gene (*ctxA*) (Hoshino *et al.*, 1998). Biotype specific characterizations were performed by using PCR assays targeted to detect *tcpA* (Classical and El Tor) (Keasler & Hall, 1993) and the type of the *rstR* (El Tor and Classical alleles) gene encoding the phage transcriptional regulator (Bhattacharya *et al.*, 2006). Mismatch amplification mutation assay (MAMA)-PCR to detect sequence polymorphism between the classical and El Tor *ctxB* gene was performed to distinguish the type of CTXB of the *V. cholerae* O1 strains included in this study (Morita *et al.*, 2008); primers and procedures were used as described previously (Alam *et al.*, 2010).

#### Antimicrobial susceptibility.

Antimicrobial susceptibility to ampicillin (AMP), ampicillin/sulbactam (AXS), nalidixic acid (NAL), ciprofloxacin (CIP), trimethoprim/sulfamethoxazole (SXT), chloramphenicol (CHL), tetracycline (TCY), levofloxacin (LVX), azithromycin (AZM), doxycycline (DOX) was determined using E-test strips (bioMérieux), according to the manufacturer’s instructions. Cut-off levels for assessing resistance were used according to CLSI document M45 guidelines (CLSI, 2010a). For nalidixic acid, the MIC break point for *Enterobacteriaceae* was used, as published in CLSI document M100 (CLSI, 2010b).

#### PFGE.

Intact agarose-embedded chromosomal DNA from 16 representative strains of *V. cholerae* O1 from Vietnam and four strains from Bangladesh and Zimbabwe was prepared and PFGE was performed using contour-clamped homogeneous electric field apparatus (Bio-Rad) according to the PulseNet procedure (Cooper *et al.*, 2006). Genomic DNA was digested with the *Not*I restriction enzyme (Gibco-BRL). The electrophoresis was performed by using CHEF-DRII system apparatus and conditions. Images were captured and converted to tif files for computer analysis (Nusrin *et al.*, 2009). *Salmonella enterica* serotype Braenderup strain H9812 was used as a molecular size marker.

## Results

### Serotyping and biotyping

Serogrouping and bio-typing of Vietnamese *V. cholerae* strains revealed that all the strains belonged to the serogroup O1 Ogawa and the biotype El Tor (Table 1). Slide agglutination tests with antisera showed that all strains were positive to polyvalent O1 and monovalent Ogawa. Biotyping showed that all strains were resistant to polymycin B 50 IU, agglutinated to chicken erythrocytes and were resistant to classical phage IV and sensitive to El Tor phage 5, which are typical traits of the El Tor biotype.

**Table 1.** Characteristics of clinical isolates of *V. cholerae* O1 EL, El Tor type; CL, classical type; +, positive; −, negative.

Country/source	Serotype	Biotype							Genotype by PCR
		Chicken cell agglutination	Polymycin B 50 U sensitivity	El Tor phage 5	Classical phage IV	*rst*R	*tcp*A	*ctx*B	
Vietnam	O1 Ogawa	+	Resistant	Sensitive	Resistant	EL	EL	CL	*omp*W^+^, *rfb*O1^+^, *rfb*O139^−^, *ctxA*^+^
Bangladesh	O1 Ogawa	+	Resistant	Sensitive	Resistant	EL	EL	CL	*omp*W^+^, *rfb*O1^+^, *rfb*O139^−^, *ctxA*^+^
Zimbabwe	O1 Ogawa	+	Resistant	Sensitive	Resistant	EL	EL	CL	*omp*W^+^, *rfb*O1^+^, *rfb*O139^−^, *ctxA*^+^
N16961 El Tor reference	O1 Inaba	+	Resistant	Sensitive	Resistant	EL	EL	EL	*omp*W^+^, *rfb*O1^+^, *rfb*O139^−^, *ctxA*^+^
O395 classical reference	O1 Ogawa	−	Sensitive	Resistant	Sensitive	CL	CL	CL	*omp*W^+^, *rfb*O1^+^, *rfb*O139^−^, *ctxA*^+^

### PCR assays

Examination of genotypic traits also confirmed that the Vietnamese *V. cholerae* were of the O1 serogroup and altered El Tor variant (Table 1). PCRs for the *V. cholerae* species-specific gene *ompW* and serogroup-specific gene *rfbO1* were positive for all strains. All strains harboured *rstR*^El^ and *tcpA* genes which are specific to strains of the El Tor biotype. Results of MAMA-PCR showed that all the *V. cholerae* isolates from Vietnam in this study harboured the *ctxB1* allele which had been identified in strains of the classical biotype worldwide and in US Gulf Coast El Tor strains. All strains were positive for the *ctxA* gene.

### Antimicrobial susceptibility

The percentages of the isolates that were susceptible, intermediately resistant or resistant to each microbial agent as well as the mean and range of MIC values are presented in Table 2. The data revealed that the isolates were susceptible to amipicillin (98 %), ampicillin/sulbactam (100 %), doxycycline (100 %), chloramphenicol (100 %), ciprofloxacin (100 %), levofloxacin (100 %), and azithromycin (95 %). However, the isolates exhibited high rate of resistance to trimethoprim/sulfamethoxazole (100 %), nalidixic acid (100 %) and tetracycline (29 %). In addition, most isolates were intermediately resistant to tetracycline (63 %).

**Table 2.** Antimicrobial susceptibility profile of *V. cholerae* O1 strains using the E-test method All MIC values are given in µg ml^−1^. nd, Not determined.

Antimicrobial(s)	No. strains	Resistance pattern						Mean MICvalues		Range of MIC values
		Sensitive		Intermediately resistant		Resistant		MIC_50_	MIC_90_	
		Percentage of strains tested	MIC	Percentage of strains tested	MIC	Percentage of strains tested	MIC			
Ampicillin	100	98	≤8	2	16	0	≥32	3	6	1–12
Ampicillin/sulbactam (2/1)	92	100	≤8/4	0	16/8	0	≥32/16	3	3	<8/4
Tetracycline	100	8	≤4	63	8	29	≥16	8	12	1–24
Doxycycline	97	100	≤4	0	8	0	≥16	2	2	0.25–3
Trimethoprim/sulfamethoxazole (1/19)	100	0	≤2/38	0	nd	100	≥4/76	32	32	>4/76
Chloramphenicol	100	100	≤8	0	16	0	≥32	0.5	0.75	0.38–1
Ciprofloxacin	100	100	≤1	0	2	0	≥4	0.38	0.5	0.125–0.75
Levofloxacin	100	100	≤2	0	4	0	≥8	0.38	0.5	0.125–0.75
Nalidixic acid*	100	0	≤16	0	nd	100	≥32	256	256	256–256
Azithromycin	100	95	≤2	4	4	1	≥8	1.5	2	0.25–32

* MIC interpretive standard for *Enterobacteriaceae*.

There were four resistance patterns (Table 3) among the 100 strains tested. The most prevalent pattern was a resistance to nalidixic acid, co-trimoxazole and tetracycline (including cases of intermediate resistance).

**Table 3.** Antibiotic resistance profiles of both intermediately resistant and resistant *V. cholerae* O1 isolates AMP, ampicillin; AXS, ampicillin/sulbactam; NAL, nalidixic acid; CIP, ciprofloxacin; SXT, trimethoprim/sulfamethoxazole; CHL, chloramphenicol; TCY, tetracycline; LVX, levofloxacin; AZM, azithromycin, DOX, doxycycline.

Type	Antibiotic resistance profile	No. strains
I	NAL, SXT	8
II	NAL, SXT, TCY	85
III	AZM, NAL, SXT, TCY	5
IV	AMP, NAL, SXT, TCY	2
Total		100

### PFGE

**Fig. 1.**
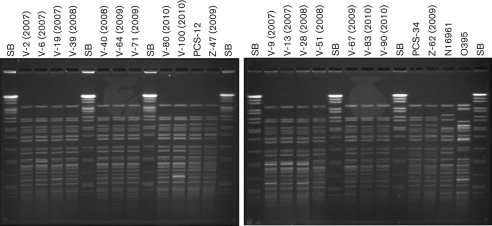
*Not*I-restricted PFGE profiles of *V. cholerae* O1 strains isolated from Vietnam, Zimbabwe and Bangladesh. Vietnamese strains are indicated with a V-number, PCS-12 and PCS34 are Bangladesh strains and Z-47(2009) and Z-62(2009) are Zimbabwe strains. SB, *Salmonella* Braenderup molecular size marker.

In the present study, PFGE analysis of the representative Vietnamese strains isolated between 2007 and 2010 showed an indistinguishable PFGE banding pattern that was typical of *V. cholerae* O1 El Tor (Fig. 1). The *Not*I restriction enzyme cleaved the chromosomal genome into 18 fragments with fragment sizes ranging from 20 to 398 kb. The PFGE patterns of isolates from Bangladesh and Zimbabwe differed slightly from the Vietnamese strains by a single band position, suggesting genetic divergence (Fig. 1).

**Fig. 2.**
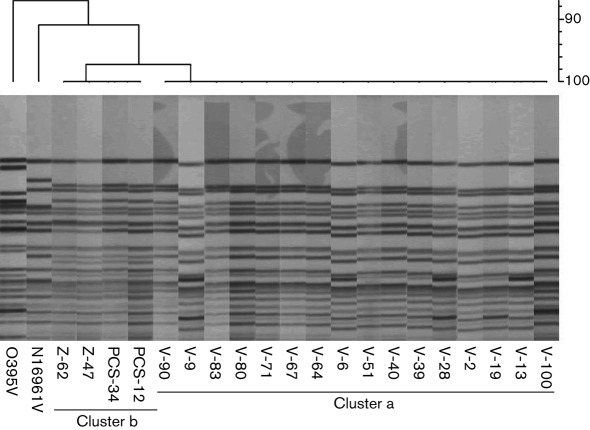
*Not*I-restricted PFGE profiles and a dendrogram reconstructed using UPGMA based on the banding patterns of *V. cholerae* O1 strains from Vietnam, Zimbabwe and Bangladesh. Cluster a includes all the Vietnamese strains showing 100 % similarity. Cluster b includes strains from Zimbabwe (Z-47 and Z-62) and Bangladesh (PCS-12 and PCS-34).

When cluster analysis was performed to produce dendrograms, prepared using the Dice similarity coefficient and unweighted pair group method with arithmetic mean (UPGMA) clustering, using the PFGE patterns, all the El Tor strains shared a major cluster (Fig. 2) in which all the Vietnamese strains shared 100 % similarity, whereas the Bangladesh and Zimbabwe strains formed another closely related cluster showing 98 % similarity to the Vietnamese strains.

The PFGE (Fig. 1) results also indicate that the altered strains from Vietnam are more closely related to the El Tor reference strain (N16961) than to the classical reference strain (O395).

## Discussion

According to the new biotyping scheme proposed for *V. cholerae* O1 (Raychoudhuri *et al.*, 2008), the results show that the Vietnamese *V. cholerae* O1 strains are variant El Tor strains. The strains have phenotypic traits of the El Tor biotype and genotypic traits of both El Tor and classical biotypes (*rstR*^El^/*ctxB1*). These variant strains were first reported in Bangladesh (Nair *et al.*, 2002, 2006). The emergence and spread of a new variant of *V. cholerae* O1 urges the redefinition of *V. cholerae* classification (Raychoudhuri *et al.*, 2008; Safa *et al.*, 2010). Recently, a new biotyping scheme was proposed, using the molecular marker genes *ctxB*, *rtxC*, *tlc*, *tcpA* and RS element, which added a further two biotypes, designated ‘hybrid’ and ‘El Tor variant’, to the classification of newly emerging *V. cholerae* O1 strains (Raychoudhuri *et al.*, 2008).

Earlier work already showed that cholera outbreaks in northern Vietnam in 2007 and early 2008 were caused by a *V. cholerae* O1 El Tor variant biotype strain producing ‘classical’ CTXB. This El Tor variant strain was probably imported into northern Vietnam around 2007 and subsequently replaced the endemic strains which contained a tandem repeat of the classical CTX prophage on the small chromosome, similar to the Mozambican variant, and were responsible for small cholera outbreaks before 2007 in Vietnam (Nguyen *et al.*, 2009). Our genotyping data reveal that cholera outbreaks in northern Vietnam between 2007 and 2010 were caused by a single clone, confirming that the new *V. cholerae* O1 El Tor variant strain is predominant and has replaced the traditional El Tor biotype.

It is currently known that all the *V. cholerae* O1 strains isolated in Bangladesh after 2001 were atypical or El Tor variant strains as they were phenotypically and genetically similar to El Tor but had the *ctxB1* gene of the classical biotype (Nair *et al.*, 2002, 2006). Subsequent studies reported such variant strains in Mozambique (Ansaruzzaman *et al.*, 2004), Vietnam (Nguyen *et al.*, 2009), Zimbabwe (this study) and Mexico (Alam *et al.*, 2010). Although the Vietnamese *V. cholerae* strains isolated between 2007 and 2010 were of the El Tor variant biotype and had PFGE patterns closely related to those of *V. cholerae* strains isolated from contemporary cholera outbreaks in Zimbabwe and Bangladesh, they had minor but consistent divergences, suggesting that the Vietnamese strains had an independent lineage and occupied a local niche.

Using multi-locus variable number tandem repeat analysis, a recent study demonstrated that altered strains from Vietnam are distant from the El Tor prototype strain responsible for the seventh cholera pandemic (Choi *et al.*, 2010). In spite of the minor divergences observed in the present study, the Vietnamese *V. cholerae* O1 strains with the RS1–CTX prophage (El Tor type *rstR* and classical *ctxB*) array on the large chromosome is likely to be more closely related to *V. cholerae* O1 El Tor variant strains currently circulating in Bangladesh (Nair *et al.*, 2006), India (Goel *et al.*, 2010) and Thailand (Okada *et al.*, 2010). However, our data are insufficient to verify the source of the *V. cholerae* O1 strains currently circulating in Vietnam.

Most of the strains from Vietnam were multi-drug resistant with common resistance to nalidixic acid, co-trimoxazole and tetracycline. All isolates were susceptible to doxycycline. Resistance to tetracycline, a common antimicrobial agent for the treatment of cholera (WHO, 2005), was also high (29 %). Both tetracycline and doxycycline belong to the tetracycline antibiotic group and cross-resistance between these agents exists (Fluit *et al.*, 2005). The use of doxycycline for cholera treatment and the trend of *V. cholerae* O1 resistance to the tetracycline group of antibiotics should be monitored. All isolates were resistant to co-trimoxazole, a recommended first-line agent for children with diarrhoea. A high incidence of co-trimoxazole resistance in *V. cholerae* O1 strains has been reported in prior studies in Africa, Asia and South America (Goel & Jiang, 2010; Ibarra & Alvarado, 2007; Mandomando *et al.*, 2007). In Vietnam, there is also an increase in resistance to co-trimoxazole among diarrhoeagenic strains of *E. coli* and *Shigella* isolated from children in Hanoi (Nguyen *et al.*, 2005). This indicates that co-trimoxazole should not be the recommended treatment for children with diarrhoea in Vietnam.

All the O1 *V. cholerae* strains were susceptible to fluoroquinolones, including ciprofloxacin and levofloxacin. However, the MIC_90_ of ciprofloxacin was close to the susceptibility breakpoint of 1 mg l^−1^. All the *V. cholerae* O1 strains from Vietnam were completely resistant to nalidixic acid (MIC 256 µg ml^−1^). *V. cholerae* O1 strains resistant to fluoroquinolones have been reported from India (Krishna *et al.*, 2006). The high incidence of nalidixic acid resistance in *V. cholerae* O1 and increased resistance of *V. cholerae* against ciprofloxacin is cause for concern (Islam *et al.*, 2009). Although the MICs of ciprofloxacin for the Vietnamese *V. cholerae* O1 strains were still below the susceptibility breakpoint, they had increased compared to historical isolates; this may be an indication of the emergence of high-level fluoroquinolone resistance in the near future. The majority of the Vietnamese strains (95 %) were still susceptible to azithromycin and this antibiotic can still be used for treatment in Vietnam, though further development of resistance needs to be monitored. Azithromycin is used for treatment of diarrhoeal diseases worldwide (de Saussure, 2009) and has shown to be effective in treatment of cholera in children and adults (Saha *et al.*, 2006). In general, the Vietnamese *V. cholerae* strains showed similar resistance patterns to Indian and Thai strains of the altered *V. cholerae* O1 El Tor biotype, carrying the classical *ctxB* allele; a new variant of *V. cholerae* O1 (Jain *et al.*, 2011; Okada *et al.*, 2010).

### Conclusion

Between 2007 and 2010, cholera outbreaks in Vietnam have been caused by a single clone of a variant *V. cholerae* O1 biotype El Tor strain. This variant was closely linked to similar variants of *V. cholerae* O1 El Tor strains isolated in Bangladesh and Zimbabwe but had a slightly divergent lineage, suggesting that they have evolved in solitary and independent ecosystem. A significant proportion of strains were multi-drug resistant, although the majority were still borderline susceptible to fluoroquinolones and susceptible to azithromycin. Empirical treatment of cholera with fluoroquinolones needs to be reconsidered.

## 
